# 

**Published:** 1914-07

**Authors:** 


### INDEX FOR VOLUME 69,

### AUGUST, 1913, to JULY, 1914

| Page | | Page | |
|---|---|---|---|
| 1— 60 | August | 403—470 | February |
| 61—132 | September | 471—538 | March |
| 133—200 | October | 539—606 | April |
| 201—268 | November | 607—674 | May |
| 269—336 | December | 675—742 | June |
| 337—402 | January | 743—810 | July |

### CONTRIBUTORS:

AARON, Charles D., Sc.D., M. D., Retention of the Gastric Contents, page 201, Nov.

AIKMAN, John, M. D., Open Air Schools as a Factor in the Prevention of Disease, page 11, Aug.

ANDREWS, L. F., M. D., Clinical Experience with Pituitrin in Obstetrics, page 614, May.

BISSELL, William G., M. D., The Wasserman Test in the Municipal Laboratory and the Interpretation to be Placed Upon Results, page 755, July.

BLAIR, Thos. S., What will be the New Standard of Morals? page 151, October.

BOWMAN, Frederick A., M. D., and James A. Mac Leod, M. D., Appendicitis, page 539, April.

BROWN, W. M., M. D., The Significance of Albuminuria to the Obstetrician, page 692, June.

BUCHANAN, J. J., M. D., Plastic Splints for General Use, with a Special Description of the Application of Leg, page 539, April.

BURKE, Joseph, M. D., Sc.D., A Contribution to the Pathology of Duodenal Ulcers, page 417, Feb.

CASE, G. M., M. D., Benefits to be Derived from Regular Medical Inspection of Our Schools, page 19, Aug.

DAVIE, Carey W., The Duties of Examiners in Lunacy, page 69, Sept.

DOOLEY, Edward M. M.D., The Phosphatic Index. Its Value to Us in the Healing Art, page 154, Oct.

EWERS, W. V., M. D., The Significance of Albuminuria in Chronic Diseases of the Alimentary System, page 685, June.

FORNET, Dr. W., Variola Vaccine in Pure Culture, page 287, Dec.

GAULTIER, Rene, Methods of Clinical Coprology as a General Indication for Diet, page 140, Oct.

GRANT, James, K. C. M. G. F. R. C. P., London, School Hygiene and Child Life, page 688, Oct.

HANES, Edward L., M. D., Rochester, N. Y., The Significance of Albuminuria in Chronic Diseases of the Nervous System, page 688, June.

HENNINGTON, C. W., B. S., M. D., Transfusion of Blood, a Modified Instrument and Tabulation of Indications, page 277, Dec.

HENNINGTON, Charles W., B. S., M. D., Gastric Disorders—From a Surgical Point of View, page 607, May.

HENNINGTON, C. W., M.D., The Significance of Albuminuria to the Surgeon, page 690, June.

KING, James E., M. D., Effect of Accident in Production of Gynecological Conditions, page 337, Jan.

LEWIS, Dr. Joseph S., An Instrument for Circumcision, page 342, Jan.

LONG, Eli H., M. D., Cardiospasm of Thirty Years' Duration, page 403, Feb.

MAC LEOD, James A., M. D., and Frederick A. Bowman, M. D., Appendicitis, page 539, April.

McGUIRE, Dr. Frank W., Treatment of Cranial Injuries, page 148, Oct.

MEISENBACH, Roland O., M. D., Some Orthopedic Conditions in the Neighborhood of the Shoulder Joint, page 410, Feb.

PARK, Roswell, A. M., LL.D., Of What Does the Universe Consist? page 471, Mar.

PLUMLEY, W. F., M. D., The Significance of Albuminuria in Chronic Diseases of the Genito-Urinary System, page 684, June.

ROACH, Dr. Walter W., Open Window Class Rooms, page 217, Nov.

SNELL, Albert C., M. D., The Significance to the Ophthalmologist, page 693, June.

SOBLE, N. W., M.D., The Significance of Albuminuria in Chronic Diseases of the Circulatory System, page 680, June.

SPANGENTHAL, Joseph, M. D., A Case of Urticaria Pigmentosa, page 76, Sept.

STEVENS, Rollin H., Further Remarks on Isotonic Sea Water in Therapeutics, page 211, Nov.

SWAN, John M., M. D., Some Cardiac Problems, page 269, Dec.

THOMPSON, W. Gilman, M. D., The Industrial Diseases, page 1, Aug.

WILLIAMS, John R., M. D., The Significance of Albuminuria in Acute Infectious Diseases, page 676, June.

WITHERSPOON, Charles R., M. D., The Chemical Tests for Albumin, page 675, June.

WRIGHT, Thew, F.A.C.S., A Case of Congenital Absence of the Vagina—Artificial Vagina Made by Intestinal Transplantation, page 283, Dec.

WHIPPLE, E. G., M. D., The Significance of Albuminuria in Chronic Diseases of the Respiratory System, page 678, June.

WOLF, Carl G. Leo-, Haemoptysis in Infants, page 743, July.

**OBITUARY:**

Apple, Dr. W., April ........ 587
Auringer, Dr. Charles, June ...... 733

Babbit, Dr. Otis H., July ........ 794
Baker, Dr. Vincent A., June ...... 732
Barrow, Dr. Frederick, Mar...... 528
Bartran, Dr. George R., Nov.... 233
Bennett, Dr. Isaac E., April..... 586
Bixby, Dr. Jesse P., May....... 656
Blanchard, Dr. Lewis, Dec....... 310
Boult, Dr. Charles E., Jan....... 370
Bradley, Dr. Allen E., Oct...... 182
Brady, Dr. John E., July........ 794
Brainard, Dr. Lucien La Bland, Jan. ........ 370
Broad, Mrs. Lucy, April......... 587
Brooks, Dr. Frank Byron, Mar.. 528
Brown, Dr. Rush P., May........ 655
Bryant, Dr. Joseph D., May..... 655
Busch, Dr. Frederick Carl, Feb.. 446

Caldwell, Dr. William, June..... 732
Carrier, Dr. Charles W., June... 732
Case, Dr. Diogenes D., Mar..... 528
Clark, Dr. Charles P., Oct....... 182
Clyde, Dr. James Dorr, Feb...... 445
Combs, Dr. Charles G., Aug...... 41
Crittenden, Dr. Sluman C., June. 733
Curtis, Dr. Newton Freeman, June ........ 733

Davis, Dr. Jacob Walter Eleeza Karr, Feb. ........ 445
Dowling, Dr. John William, July 794
Dennis, Dr. Bernard F., Oct..... 183
De Nike, Dr. George Henry, Aug. 41
Doyle, Dr. Gregory, Sept........ 103
Duhring, Dr. Louis A., Sept..... 103
Durand, Dr. Charles F., Dec...... 310

Edwards, Dr. George A., Jan..... 370
Ellis, Dr. Lucien E., Sept........ 105
Emmett, Dr. James O., June..... 732
Ennis, Dr. Alexander, April..... 587

Flood, Dr. Henry, Feb........... 445

Goodyear, Dr. Miles D., Feb..... 444
Granger, Dr. James, Feb......... 445

Hallock, Dr. Harry M., Aug....... 41
Hamill, Dr. Vincent G., May.... 656
Harris, Dr. Sally A., Nov........ 233
Hawley, Dr. William H., June... 733
Head, Augustus Philemon, M. D., Aug. ........ 41
Heldrich, Dr. Philip, July........ 794
Hicks, Dr. Walter Scott, May.... 655
Hopkins, Dr. Henry Enos, Dec... 310

Hopkins, Dr. Ira D., April....... 587
Hotaling, Dr. Albert Steuben, Oct. ........................ 182
Howell, Dr. Harry B., April..... 587
Hoyt, Dr. Eugene F., Aug. ...... 41
Hoyer, Dr. John B., May........ 656
Hunt, Dr. James Gillespie, July.. 794
Hyde, Dr. Edward Everett, Aug.. 41

Jacobson, Dr. Nathan, Oct....... 183
Jones, Dr. Ira L., Oct............ 182
Kiersted, Dr. Charles Francis, June ........................ 733
Kinne, Dr. George Rite, June.... 732
Knapp, Dr. Frank, Mar.......... 528

Lavenson, Dr. R. S., Sept........ 105
Le Fevre, Dr. Egbert, May...... 655
Leonard, Dr. Charles Lester, Nov. 233
Lester, Dr. L. B., Nov........... 233
Lewis, Dr. Frederick Llewellyn, June ........................ 732
Linn, Dr. W. T., Sept............ 105

Mc Burney, Dr. Charles, Dec..... 310
McDonnell, Dr. John Archibald, Nov. ........................ 233
Mitchell, Dr. S. Weir, Feb........ 433
Myer, Dr. Jesse S., Dec.......... 311

Neff, Dr. J. B., Sept............. 103
Newman, Dr. Omar E., Nov...... 233

Park, Dr. Roswell, Mar......... 513
Parker, Dr. William H., April.... 586
Parsons, Dr. Ralph Lyman, April 586
Patterson, Dr. William Rex, Nov. 233
Pierce, Dr. Ray Vaughan, Mar... 528
Pierce, Dr. Sidney A., Oct....... 182

Reames, Dr. Edward C., Dec..... 310

Seaman, Dr. Harry F., June..... 732
Sheldon, Dr. A. F., Feb.......... 445
Spitzka, Dr. Edward Charles, Feb. ........................ 446
Stearns, Dr. George Reynolds, Sept. ........................ 104
Stearns, Dr. Phineas Sewall, Aug. ........................ 40
Sutton, Dr. C. R., Nov........... 233

Taplin, Dr. Mortimer M., Oct.... 182
Torney, Brigadier General George Henry, Feb. .................. 445
Tron, Dr. Stanley Emanuele, Sept. ........................ 105
Troup, Dr. Alexander M., Oct.... 183

Wadsworth, Dr. Robert, Nov.... 234
Warren, Dr. Stephen G., Nov.... 233
Wathen, Dr. William H., Dec.... 310
Watt, Dr. James Lewis, Aug...... 41
Wells, Dr. Emily H., Oct......... 182
Williams, Dr. James Samuel W., Sept. ........................ 103
Wyckoff, Dr. George S., June.... 733

Zielly, Dr. Henry E., Mar........ 528

GENERAL INDEX

ABORTION Bill, page 505, Mar.

Accident, Effect of in Producing Gynaecologic Conditions, James E. King, page 337, Jan.

Advertising Ethics and Diabetes, Edit., page 490, Mar.

Ages, Distribution of Population by, Edit., page 160, Oct.

Albuminuria, Symposium, page 675, June.

Alcohol, Tuberculosis and Venereal diseases as Factors in Stirpiculture, Edit., page 28, Aug.

American College of Physicians, page 307, Dec.; page 353, Jan.; page 432, Feb.

Anaesthesia among Indians, J. M. C. Kukay, page 87, Sept.

Anaesthesia, Rectal, page 365, Jan.

Animal Experimentation, page 431, Feb.

Animals Killed for Food in U. S., 1912, page 37, Aug.

Antivivisectionists, Hints from, Edit., page 424, Feb.

Appendicitis, J. A. MacLeod and F. A. Bowman, page 539, April.

A Question of Time, Edit., page 782, July.

Artificial Vagina, Thew Wright, page 283, Dec.

Automobile; Census, page 575, April; Comparative Danger, page 574, April; Fractures, page 505, Mar.; Notes, page 569, April.

BALKAN Wars, Mortality, page 305, Dec.

Benefits to be Derived from Regular Medical Inspection of Schools, G. M. Case, page 19, Aug.

CARDIAC Problems, John M. Swan, page 269, Dec.

Cardiospasm of Thirty Years Duration, Eli H. Long, page 403, Feb.
Centenarians, page 34, Aug.; page 298, Dec.; page 358, Jan.
Charity, Misplaced, Edit., page 427, Feb.
Circumcision, Instrument for, Joseph S. Lewis, page 342, Jan.
Cocaine Law, page 172, Oct.
Collegiate Year in Medicine, Edit., page 82, Sept.
Congenital Absence of Vagina, Artificial Vagina, Thew Wright, page 283, Dec. (See page 296, Dec.)
Contract Practice, A Desirable Kind, Edit., page 219, Nov.
Constitutionality of Legislation Restricting Medical Practice, Edit., page 699, June.
Contribution to Pathology of Duodenal Ulcers, Joseph Burke, page 417, Feb.
Coprology, Methods of Clinical, as General Indication for Diet., Rene Gaultier, page 140, Oct.
Cranial Injuries, Treatment of, Frank W. McGuire, page 748, Oct.

DASHEEN, page 352, Jan.
Death Rate, Standardized, States and Cities, page 428, Feb. In N. Y. City and Rural N. Y., chart, page 581, Apr.
Deaths from Violence, Edit., page 160, Oct.
Diabetes and Advertising Ethics, page 490, March.
Dispensary Abuse, Edit., page 294, Jan.
Distribution of Population by Ages, Edit., page 160, Oct.
Duodenal Ulcer, Joseph Burke, page 417, Feb.
Duties of Examiners in Lunacy, Carey W. Davie, page 69, Sept.

EDUCATION, Edit., page 220, Nov.; Medical, page 365, Jan.
Eugenics, See "Alcohol, Tuberculosis, etc.," Edit., page 28, Aug.; Eugenics and Sentiment, Edit., page 158, Oct.
European Travel, page 702, June.

FACTS, Wanted a Bureau of, Edit., page 494, March.
Fetichism and Mystery, page 567, Apr.

GASTRIC CONTENT, Retention of, Charles D. Aaron, page 201, Nov.
Gastric Disorders from a Surgical Point of View, Charles W. Hennington, page 607, May.
Genealogy, Edit., page 493, March.
Gettysburg Anniversary, Notes on, page 30, Aug.
Gynaecologic Conditions, Effects of Accident in Production of, James E. King, page 337, Jan.

HAEMOPTYSIS In Infants, Carl G. Leo-Wolf, page 743, July.
High Cost of Living, Edit., page 628, May.
House of Delegates of A. M. A., Legality of, page 300, Dec.
Hydrophobia Germ, page 437, Feb.
Hygiene, Congress on School, Edit., page 25, Aug.
Hygienic and Sanitary Publicity, Edit. page 294, Jan.

IMMODEST Dress, Edit., page 566, Apr.
Income Tax, Edit., page 345, Jan.
Independent Medical Journals and Official Organs, Edit., page 218, Jan.
Indians, Anaesthesia Among, J. M .G. Kukay, page 87, Sept.
Industrial Diseases, William Gilman Thompson, page 1, Aug.
Infantile Paralysis, page 230, Nov.
Insanity Law of N. Y., page 95, Sept.
Ipso Facto or Permanent Membership, Edit., page 563, Apr.
Isotonic Sea Water in Therapeutics, Further Remarks, Rollin H. Stevens page 211, Nov.

LAWS of N. Y., Cocaine, page 172, Oct.; Insanity, page 95, Sept.
Lunacy, Duties of Examiners, Carey W. Davie, page 69, Sept.

METHODS of Clinical Coprology as a General Indication for Diet, Rene Gaultier, page 140, Oct.
Mortality, See Death Rate.
Moving Pictures, Edit., page 26, Aug.
Mystery and Fetichism, Edit., page 567, Apr.

NATIONAL Dental Association, Edit., page 702, June.
Nurse, Bill to Restrict the Term, page 504, March.

OPEN AIR Schools as a Factor in the Prevention of Disease, John Aikman, page 11, Aug.
Open Window Class Rooms, Walter W. Roach, page 217, Nov.
Orthopaedic Conditions in Neighborhood of Shoulder Joint, Roland O. Meisenbach, page 410, Feb.

PARK, Roswell, Edit., page 489, Mar.
Personal Liberty, Edit., page 779, July.
Phosphatic Index, E. M. Dooley, page 154, Oct.
Pituitrin in Obstetrics, L. F. Anderson, page 614, May.
Plaster Splints for General Use, J. J. Buchanan, page 63, Sept.
Population, Distribution by Ages, Edit., page 160, Oct.
Prolongation of Life, Statistics, page 87, Sept.
Protect the Little Boy, Edit., page 25, Aug.
Psychic Depression, Can it Cause Death?, Edit., page 700, June.

QUACKS, How do they get their Address Lists?, Edit., page 220 Nov.

RETENTION of Gastric Contents, Charles D. Aaron, page 201, Nov.

SANITATION, A Neglected Detail, Edit., page 350, Jan.
Schools, Benefits from Medical Inspection, G. M. Case, page 19, Aug.; Open Air, John Aikman, page 11, Aug.
School Hygiene and Child Life, James Grant, page 133, Oct.
Sea Water, Isotonic, Rollin H. Stevens, page 211, Nov.
Segregation, Edit., page 422, Feb.
Shoulder Joint, Some Orthopaedic Conditions in Neighborhood of, Roland O. Meisenbach, page 410, Feb.
Social Reform, One Obstacle to, Edit., page 293, Dec.
Socialism, Hint from, Edit., page 294, Dec.
Spelling, Standards Adopted by Buffalo Medical Journal, page 164, Oct.
Splints, Plastic, J. J. Buchanan, page 63, Sept.
Surgeons, American College of, page 307, Dec.; page 353, Jan.; page 432, Feb.

THE TOUCH, Edit, page 625, May.
Transfusion of Blood, Modified Instrument and Tabulation of Indications, Charles W. Hennington, page 277, Dec.
Tuberculosis, Is the Bacillus the Cause of, Edit., page 290, Dec.; Hospitals, page 429, Feb.; Mortality Chart, page 498, March; Turtle Tuberculin, Edit., page 701, June.
Typhoid Fever, Edit., page 78, Sept.; and Tuberculosis, Edit., page 701, June; Vaccination, page 437, Feb.; page 721, June.
Twelfth International Ophthalmic Congress and Antisemitism, page 573, Apr.

UGLY DUCKLINGS of Medical Dialect, Edit., page 83, Sept.
Universe, Of What Does the Universe Consist, Roswell Park, page 471, March.
Urticaria Pigmentosa, Joseph Spangenthal, page 76, Sept.

VAGINA, Congenital Absence, Artificial Vagina, Thew Wright, page 283, Dec.
Variola Vaccine in Pure Culture, W. Fornet, page 287, Dec.
Vital Statistics of N. Y. State., page 571, April (See Death Rates)
Vivisection and Actual Facts, Edit, page 696, June. (See Animal Experiment, Anti-)

WASSERMANN Reaction, Wm. G. Bissell, page 755, July.
Weight Fallacies, Edit., page 776, July.
Wholesome Imperfection, Edit, page 627, May.
Will There be a New Standard of Morals, Thomas S. Blair, page 151, Oct.
Wood Alcohol, page 295, Dec.; page 306, Dec.; page 359, Jan.

